# Supplementary material for: Actomyosin and the MRTF-SRF pathway downregulate FGFR1 in mesenchymal stromal cells
Source: Commun Biol. 2020 Oct 16;3:576. doi: 10.1038/s42003-020-01309-1 (PMC7567845; doi:10.1038/s42003-020-01309-1)
Supplement: Supplementary file 1 — Supplementary Information [file 42003_2020_1309_MOESM1_ESM.pdf]

Supplementary Information

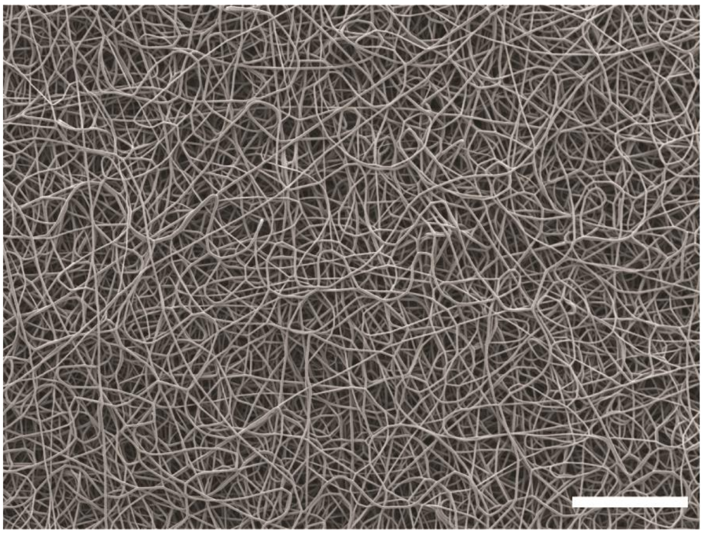

Supplementary Figure 1. Overview of microfibrillar substrate. Scalebar 100 µm.

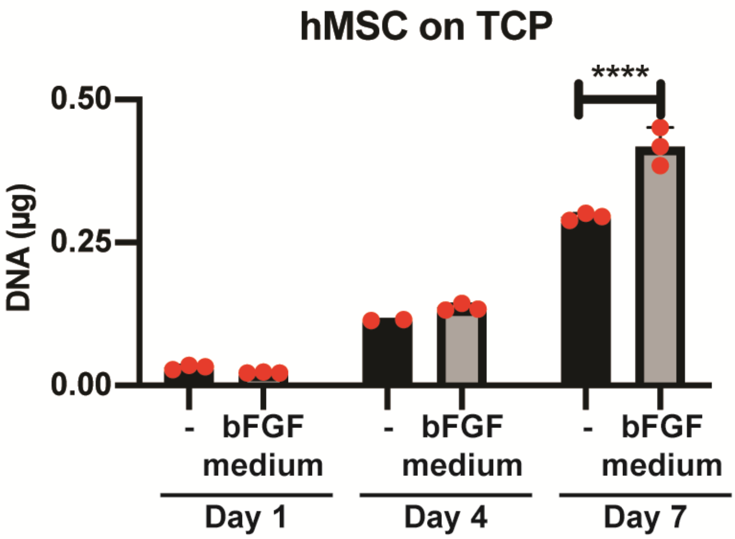

Supplementary Figure 2. Increased proliferation of hMSCs in response to bFGF. DNA quantification of hMSCs cultured on TCP in basic medium (-) or basic medium + 10 ng/ml bFGF harvested on day 1, 4 or 7. n=3 for each condition. One-way ANOVA with Tukey's post-hoc test. **** $p<0.0001$. Error bars indicate mean±SD. Individual data points as red dots.

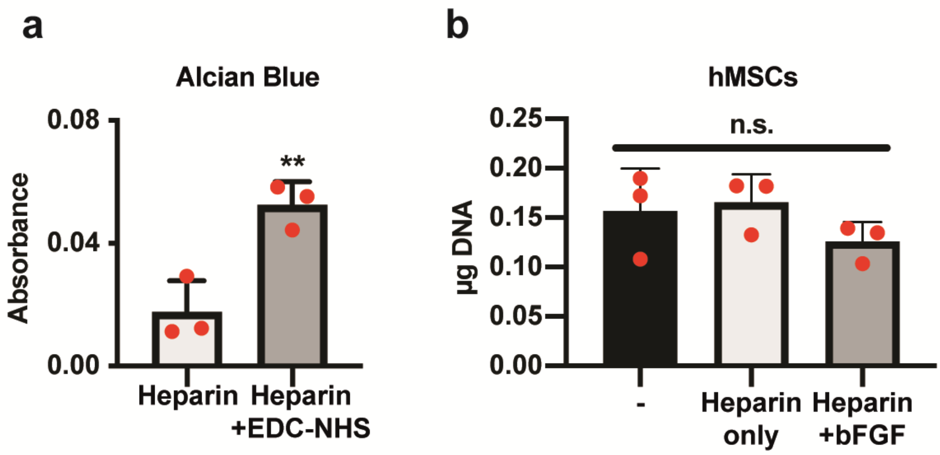

Supplementary Figure 3. No effect heparin bound bFGF on hMSC proliferation. a, Alcian blue analysis of heparin bound to microfibrillar substrates with incorporated PEG-NH_2 by EDC-NHS chemistry, or aspecifically absorbed heparin, without EDC-NHS. Student's t-test. ** $p<0.01$. **b,** DNA quantification of hMSCs cultured for 7 days on unfunctionalized microfibrillar PEG-NH_2 substrates (-), or microfibrillar PEG-NH_2 substrates functionalized with heparin and with or without absorbed bFGF. Kruskal-Wallis with post-hoc test. n.s. $p>0.05$. **a, b,** n=3 for each condition. Error bars indicate mean±SD. Individual data points as red dots.

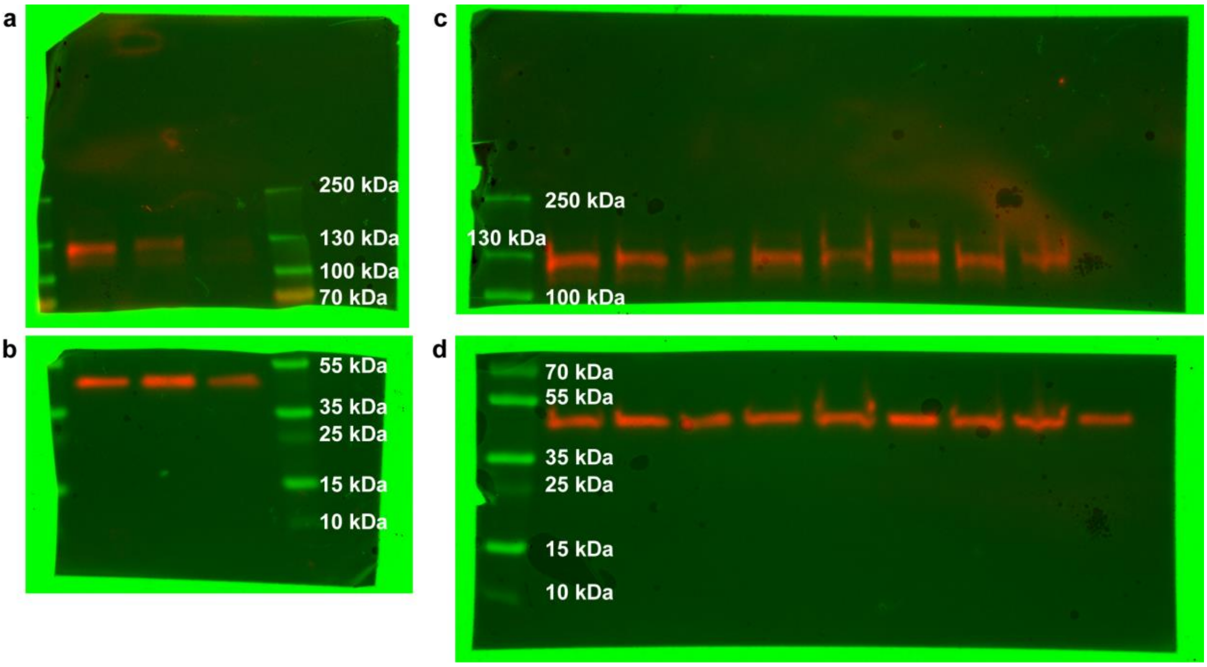

Supplementary Figure 4. Full unedited blots of figure 2. FGFR1 **(a)** and TBP **(b)** of the hMSC blot. FGFR1 **(c)** and TBP **(d)** of the Fibroblast blot. Including the ladder, lanes 4, 5 and 6 were used in figure 2.

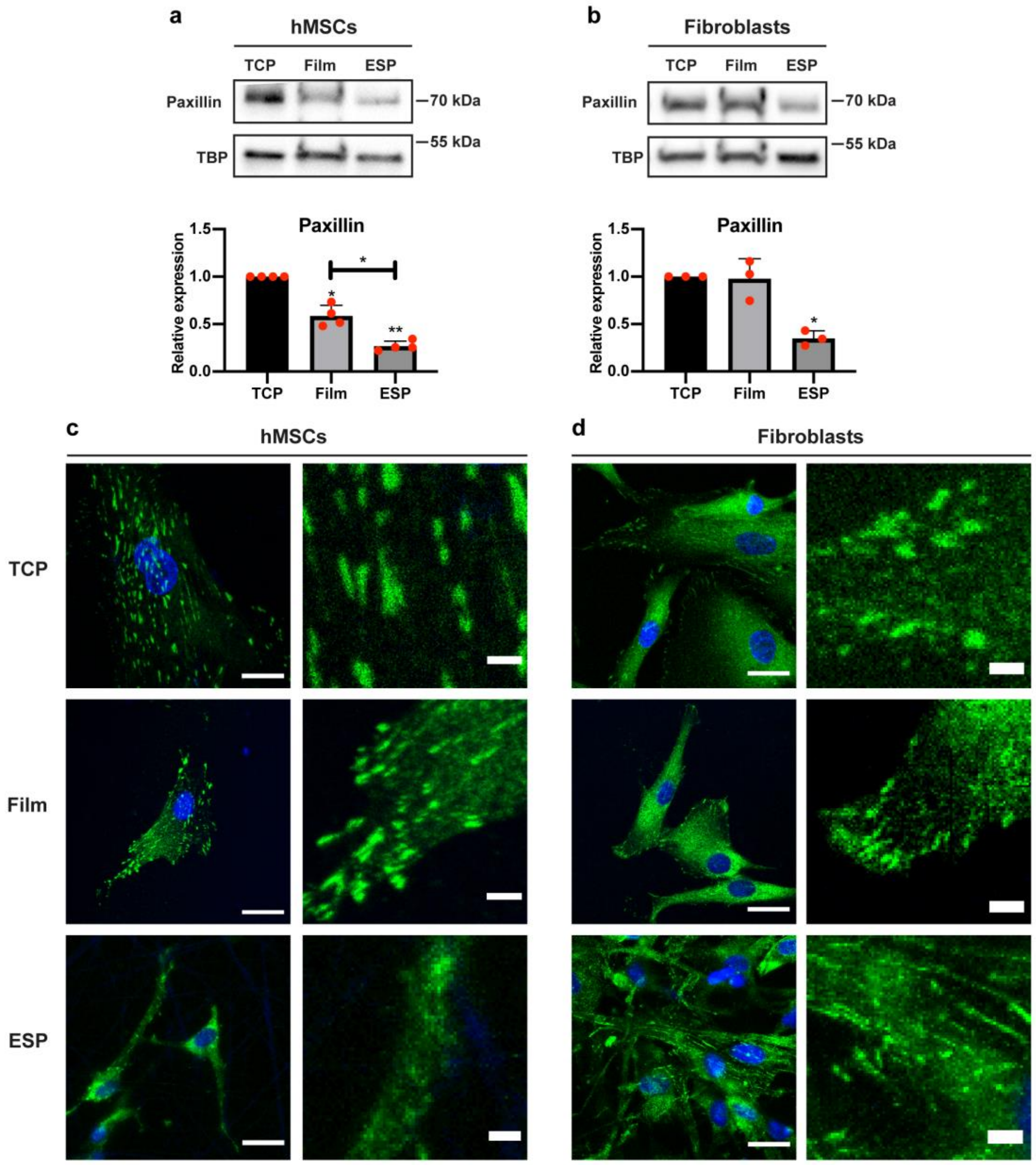

Supplementary Figure 5. Reduced paxillin expression on microfibrillar substrates. a, b, Western blot of paxillin and TBP (as loading control) of hMSCs **(a)** or human dermal fibroblasts **(b)** on TCP, films or microfibrillar substrates (ESP). Graphs depict quantification of western blots of paxillin/TBP from 4 **(a)**, or 3 **(b)** independent experiments, normalized to TCP. Stars above bars indicate significance compared to TCP. Repeated measures ANOVA with Tukey's post-hoc test. * $p<0.05$; ** $p<0.01$. Error bars indicate mean±SD. Individual data points as red dots. **c, d,** Representative images of hMSCs **(c)** or human dermal fibroblasts **(d)** stained for paxillin (green) and nuclei (blue) on TCP, films or microfibrillar substrates (ESP). Right panels represent a 5x magnification of the respective left panel. Scalebars represent 25 µm (left panels) and 4 µm (right panels).

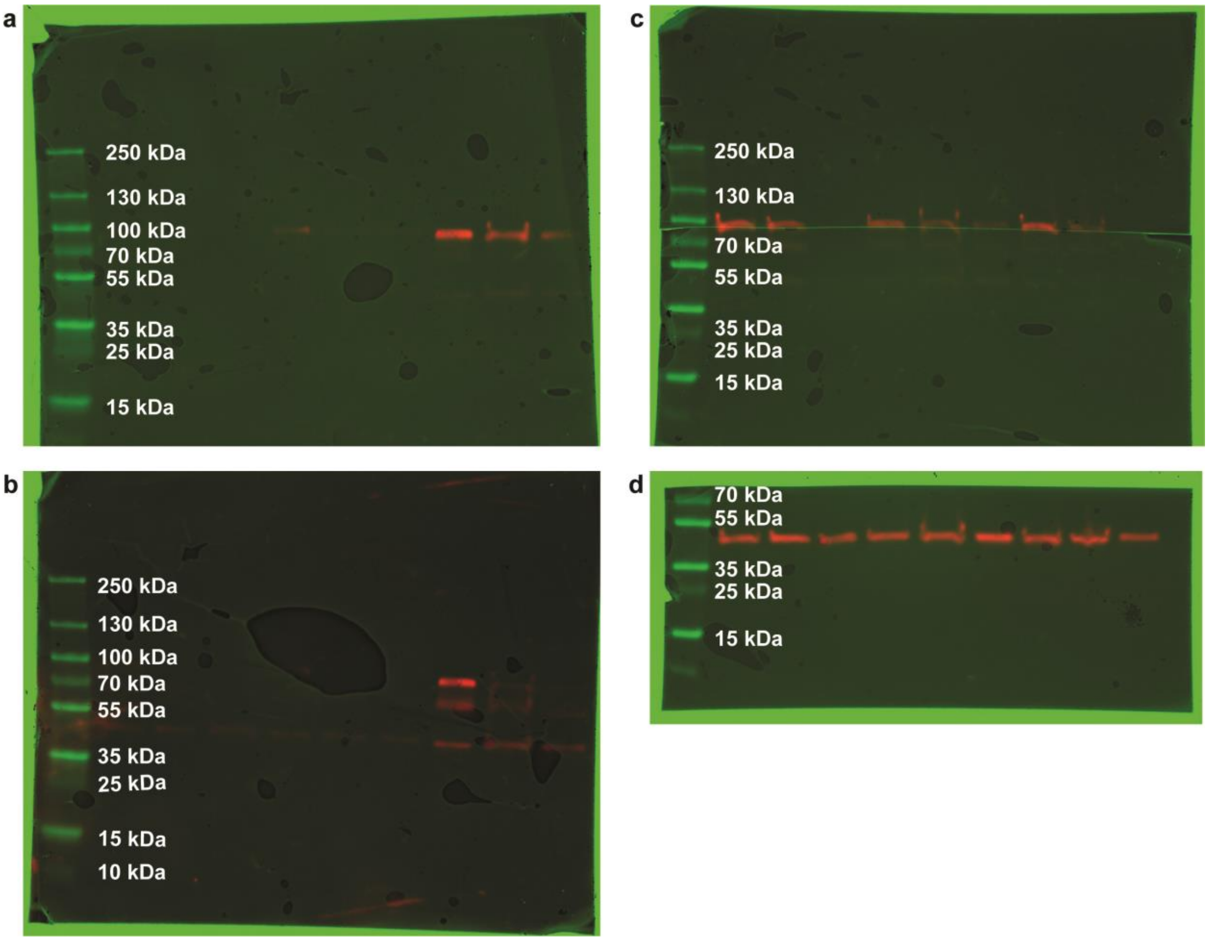

Supplementary Figure 6. Full unedited blots of figure 3. Zyxin **(a)** and TBP **(b)** of the hMSC blot. The most right three lanes were used in figure 3. The Zyxin **(a)** blot was incubated with the TBP antibody, resulting in the TBP blot **(b)**. The TBP band is visible between 35 and 55 kDa. The Zyxin band is still visible around 100 kDa. The extra band around 70 kDa is the band from another antibody. All antibodies were carefully checked for extra bands or interference before such serial antibody incubations were performed. Zyxin **(c)** and TBP **(d)** of the Fibroblast blot. Only the bottom half of the blot was incubated with TBP, resulting in the TBP blot **(d)**. Including the ladder, lanes 4, 5 and 6 were used in figure 3.

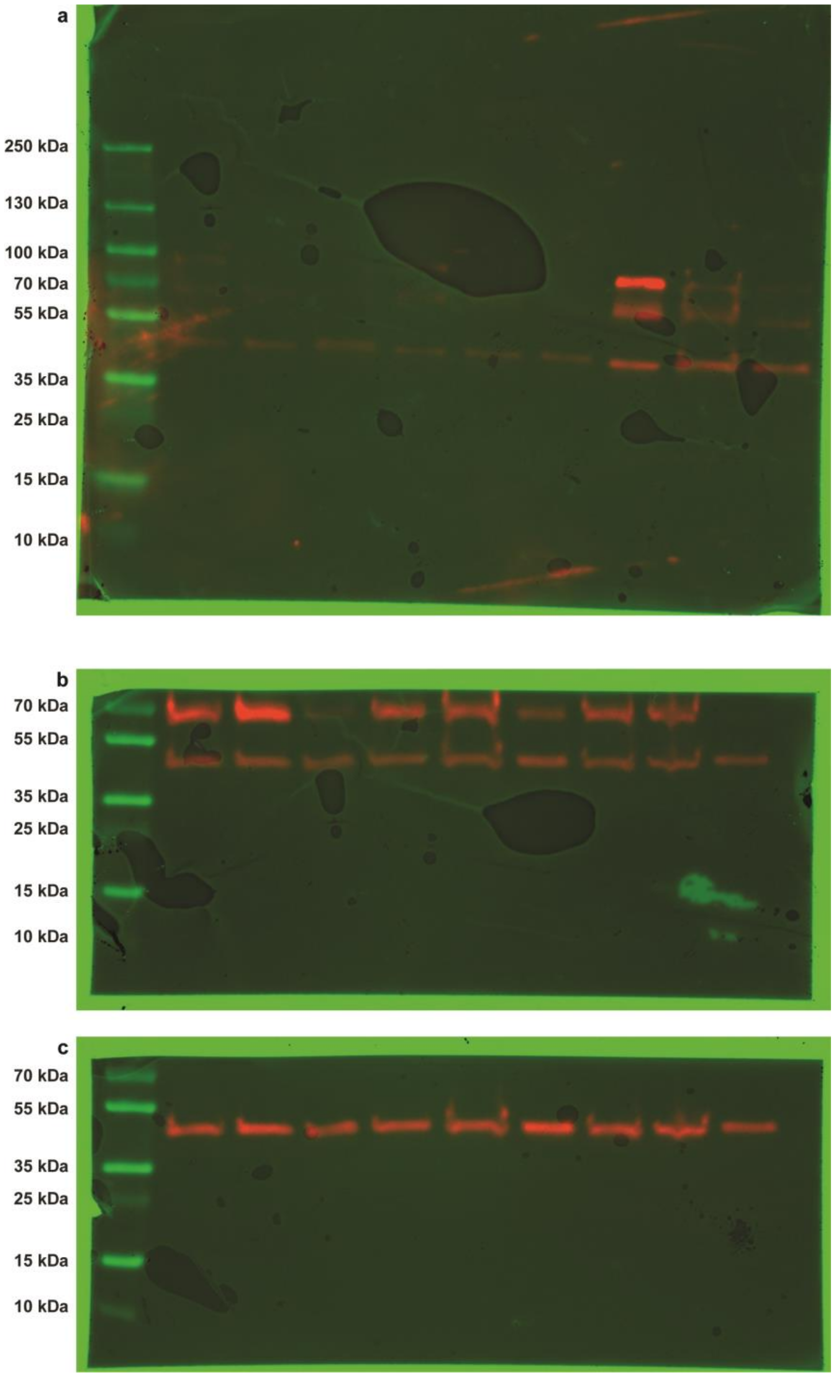

Supplementary Figure 7. Full unedited blots of supplementary figure 5. Paxillin and TBP **(a)** of the hMSC blot. The blot was incubated with paxillin and TBP at the same time. The lowest band is the TBP band, while the middle band is the paxillin band. The top band is from another previously incubated antibody. The last three lanes were used for the figure. All antibodies were carefully checked for extra bands or interference before such serial antibody incubations were performed. Paxilin **(b)** and TBP **(c)** of the fibroblast blot. The blot was first incubated with TBP **(c)** and the following day with paxillin **(b)**. Including ladder, lanes 5, 6 and 7 were used for the figure.

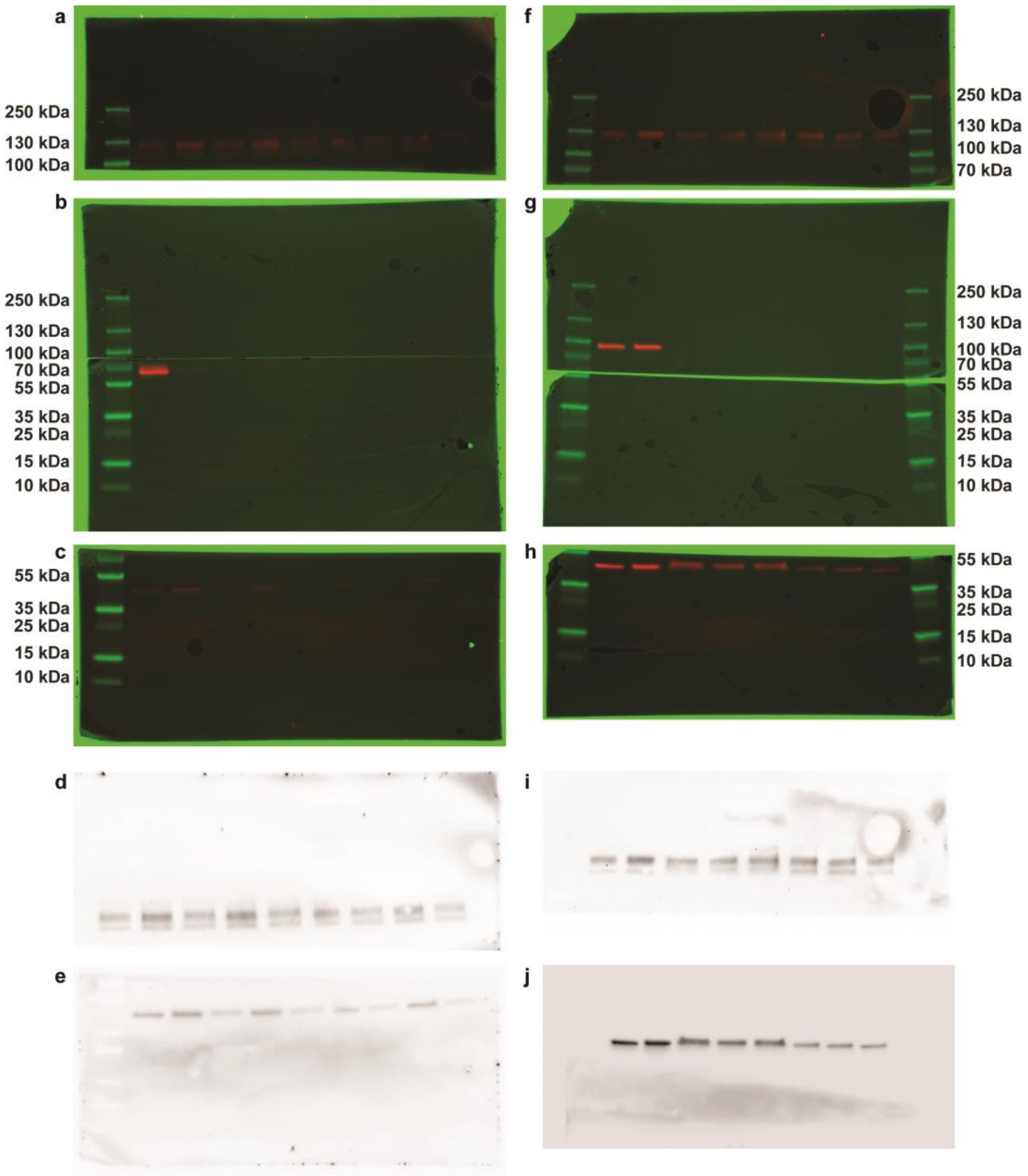

Supplementary Figure 8. Full unedited blots of figure 4. FGFR1 **(a)**, paxillin **(b)** and TBP **(c)** of the PXN-KD blot. The top part of the blot was incubated with FGFR1 antibodies and the bottom with TBP antibodies, the following day both parts were incubated with paxillin antibodies. Including ladder, lane 2, 3 and 4 were used in figure 4. The FGFR1 **(d)** and TBP **(e)** blots are shown without the colorimetric image to more clearly display the bands. FGFR1 **(f)**, zyxin **(g)** and TBP **(h)** of the ZYX-KD blot. The top part of the blot was incubated with FGFR1 antibodies and the bottom with TBP antibodies, the following day both parts were incubated with zyxin antibodies. Including ladder, lane 2, 5 and 7 were used in figure 4. The FGFR1 **(i)** and TBP **(j)** blots are shown without the colorimetric image to more clearly display the bands. All antibodies were carefully checked for extra bands or interference before such serial antibody incubations were performed.

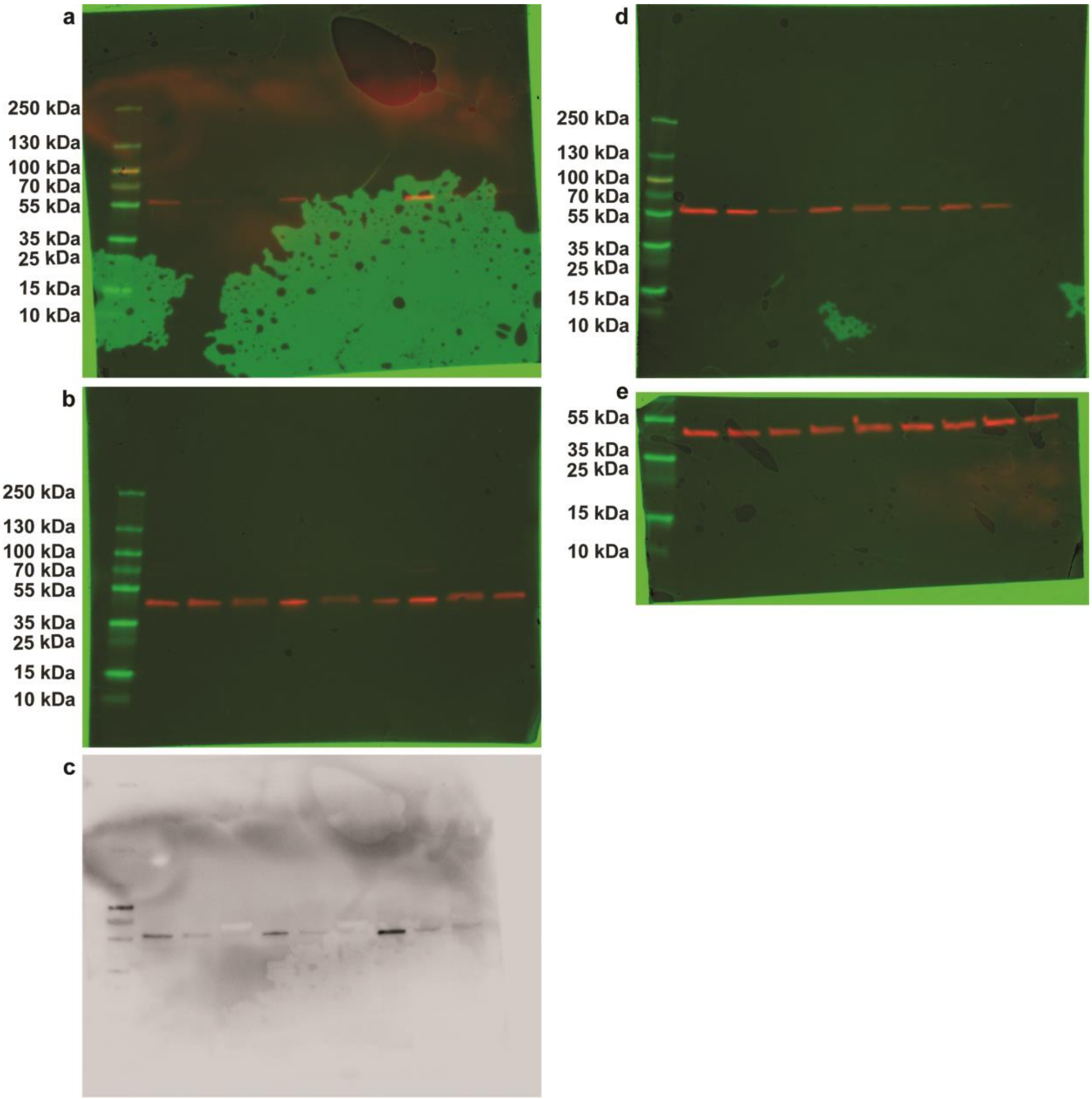

Supplementary Figure 9. Full unedited blots of figure 5. SRF **(a)** and TBP **(b)** of the hMSC blot. SRF only, without the colorimetric image, to more clearly display the bands **(c)**. Because the TBP and SRF bands were too close together and required different buffers, the samples were run on different blots. Relative TBP expression was compared to previous runs with these samples to ensure reliable results. Including ladder, lane 2, 3 and 4 were used for figure 5. SRF **(d)** and TBP **(e)** of the Fibroblast blot. Including the ladder, lanes 4, 5 and 6 were used in figure 2.

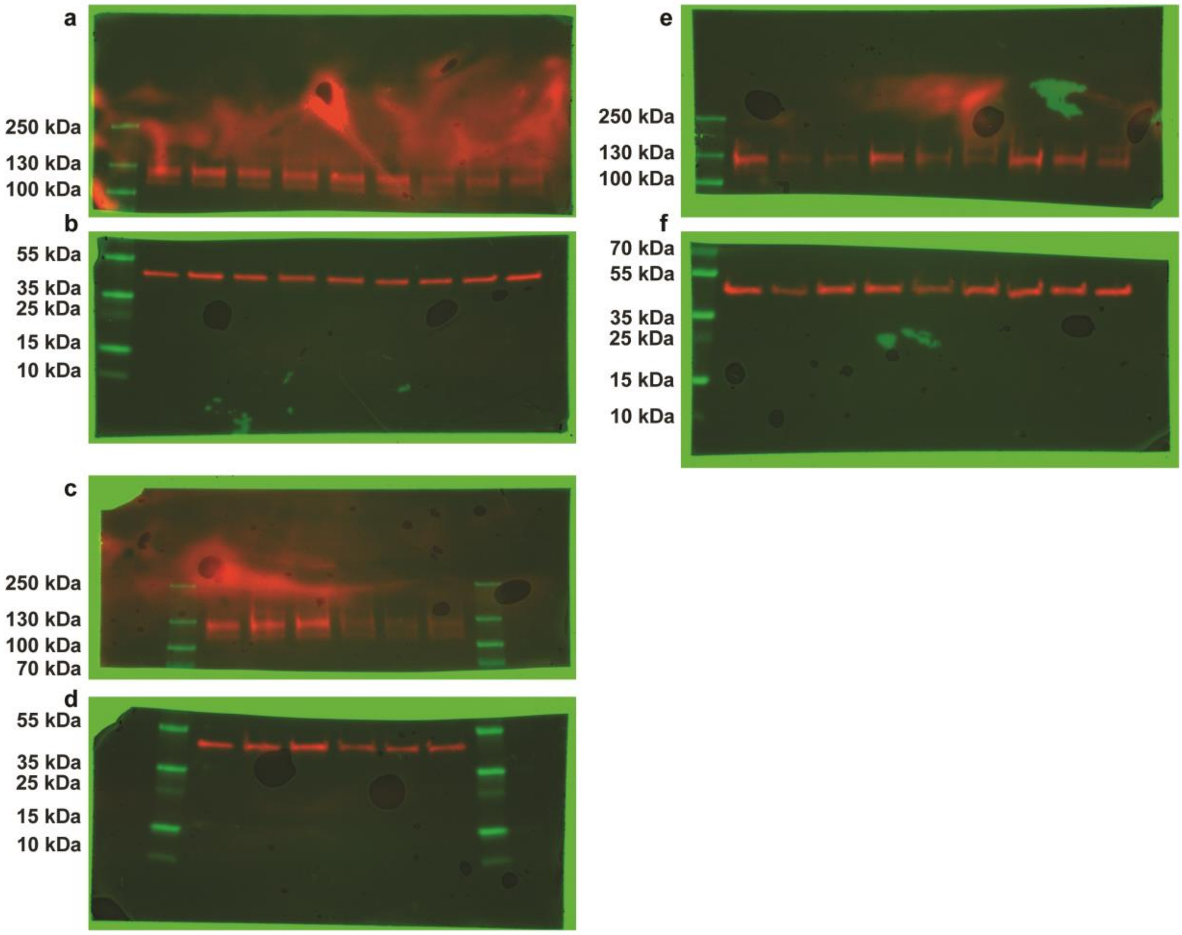

Supplementary Figure 10. Full unedited blots of figure 6. FGFR1 **(a)** and TBP **(b)** of the hMSC MRTF/SRF inhibitor blot. Including the ladder, Lanes 3 and 9 were used for figure 6. FGFR1 **(c)** and TBP **(d)** of the hMSC blebbistatin blot. Including the ladder, Lanes 2 and 5 were used for figure 6. FGFR1 **(e)** and TBP **(f)** of the fibroblast MRTF/SRF inhibitor blot and the fibroblast blebbistatin blot. Including the ladder, Lanes 2 and 4 were used for the fibroblast MRTF/SRF inhibitor panel in figure 6 and lanes 5 and 6 for the fibroblast blebbistatin panel in figure 6.

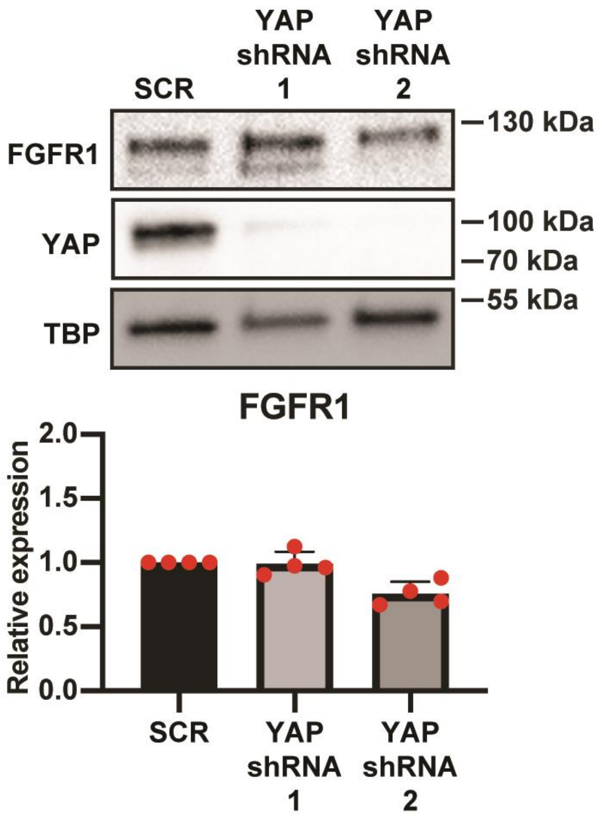

Supplementary Figure 11. YAP does not regulate FGFR1 expression. a, b, Western blot of FGFR1, YAP and TBP (as loading control) of hMSCs transduced with YAP shRNA, cultured on TCP. Graphs depict quantification of western blots of FGFR1/TBP from 4 biological replica's, normalized to TCP. Error bars indicate mean±SD. Repeated measures ANOVA with Tukey's post-hoc test. Individual data points as red dots.

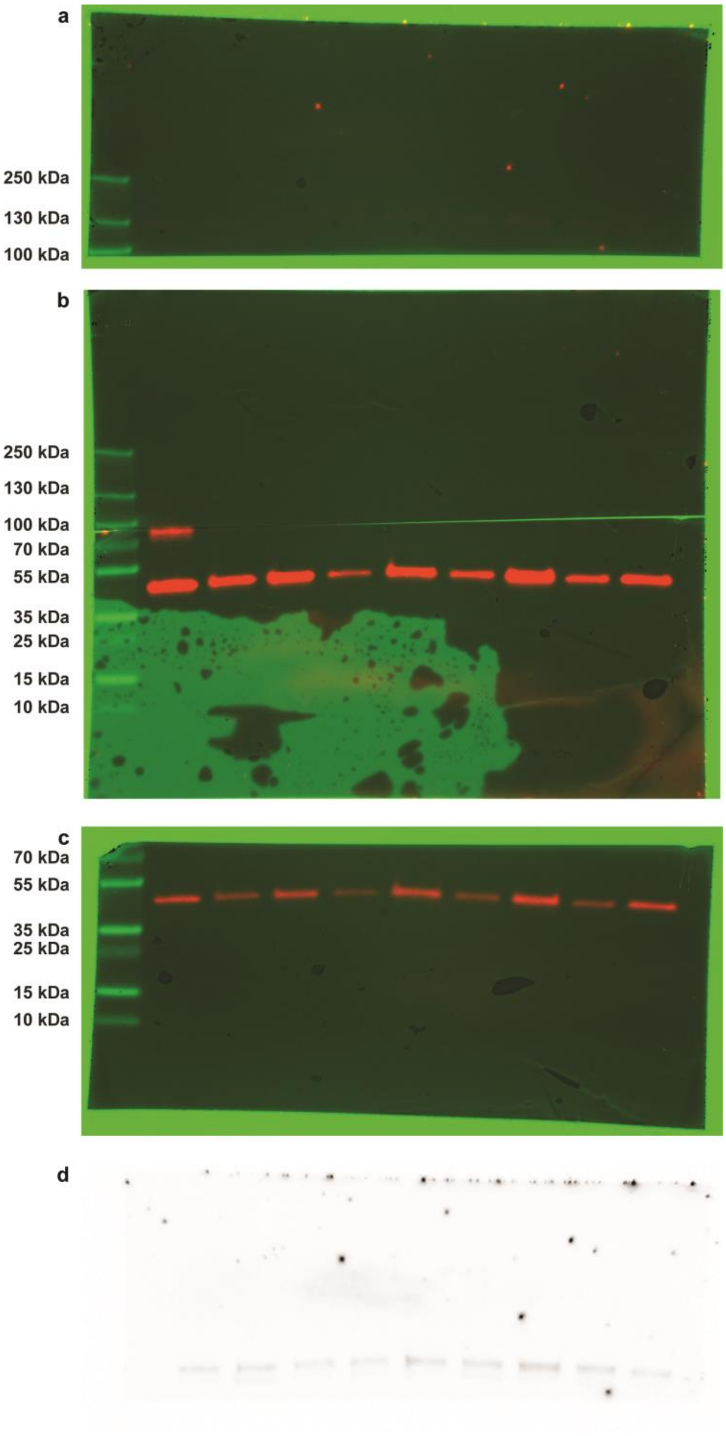

Supplementary Figure 12. Full unedited blots of supplementary figure 11. FGFR1 **(a)**, YAP **(b)** and TBP **(c)** of the YAP knockdown blot. The top part of the blot was incubated with FGFR1 antibodies **(a)**, the bottom with TBP antibodies **(c)** and both parts were then incubated with YAP antibodies **(b)**. The bottom bands in the YAP blot **(b)** are the bands from the TBP blot. All antibodies were carefully checked for extra bands or interference before such serial antibody incubations were performed. FGFR1 is displayed without the colorimetric image to more clearly show the bands **(d)**. Including ladder, lanes 2, 3 and 4 were used for the figure.

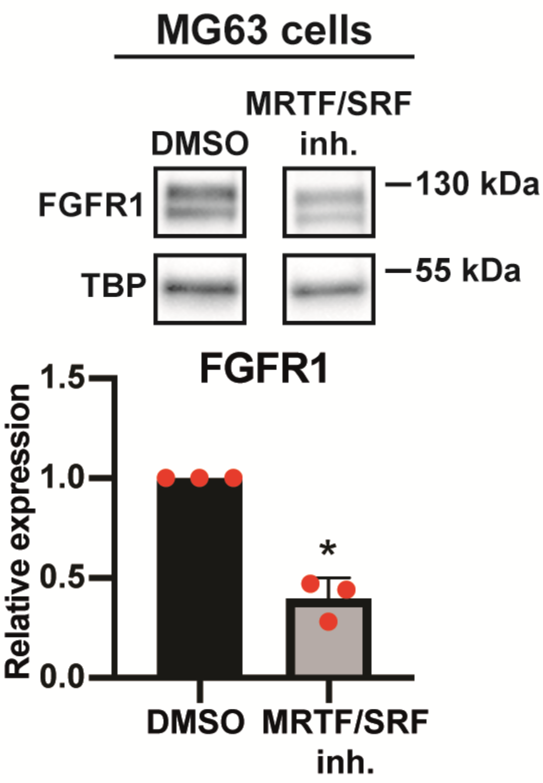

Supplementary Figure 13. MRTF/SRF regulates FGFR1 in MG63 cancer cells. Western blot of FGFR1 and TBP (as loading control) of MG63, an osteosarcoma cell-line, cultured on TCP and treated with MRTF/SRF inhibitor CCG203971. Graph depicts quantification of western blots of FGFR1/TBP from 3 biological replicas, normalized to TCP. Individual data points as red dots.

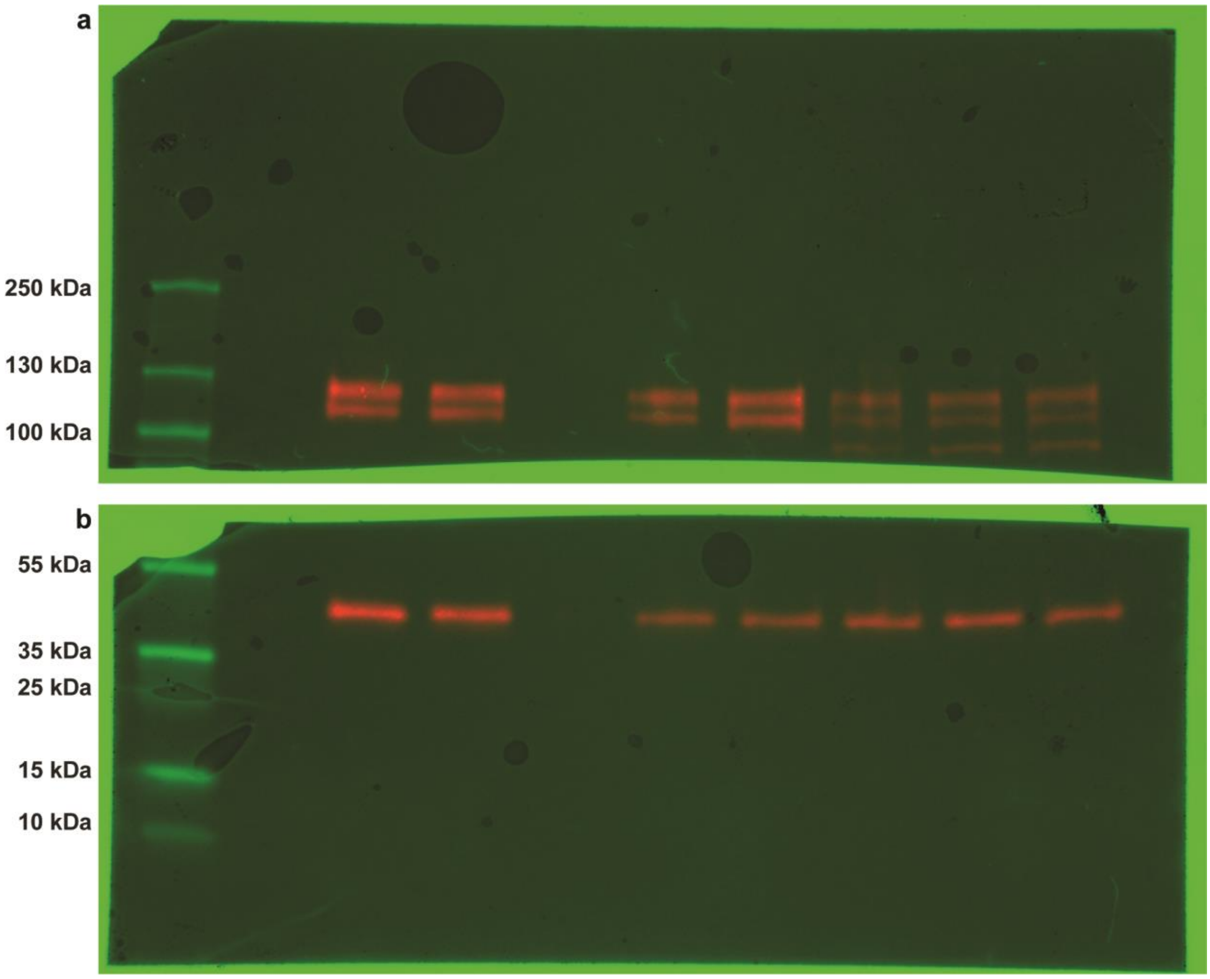

Supplementary Figure 14. Full unedited blots of supplementary figure 13. FGFR1 **(a)** and TBP **(b)** of MG63 blot. Including ladder, lanes 3 and 9 were used for the figure.
