# Supplementary material for: Actomyosin and the MRTF-SRF pathway downregulate FGFR1 in mesenchymal stromal cells
Source: Commun Biol. 2020 Oct 16;3:576. doi: 10.1038/s42003-020-01309-1 (PMC7567845; doi:10.1038/s42003-020-01309-1)
Supplement: Supplementary file 2 — Description of Additional Supplementary Files [file 42003_2020_1309_MOESM2_ESM.docx]

Description of additional Supplementary files

Supplementary Data 1: Source data underlying the graphs in the paper.
