# Supplementary material for: Actomyosin and the MRTF-SRF pathway downregulate FGFR1 in mesenchymal stromal cells
Source: Commun Biol. 2020 Oct 16;3:576. doi: 10.1038/s42003-020-01309-1 (PMC7567845; doi:10.1038/s42003-020-01309-1)
Supplement: Supplementary file 4 — Reporting Summary [file 42003_2020_1309_MOESM4_ESM.pdf]

nature research

Corresponding author(s): Lorenzo Moroni

Last updated by author(s): 26-2-2020

Reporting Summary

Nature Research wishes to improve the reproducibility of the work that we publish. This form provides structure for consistency and transparency in reporting. For further information on Nature Research policies, see Authors & Referees and the Editorial Policy Checklist .

Statistics

For all statistical analyses, confirm that the following items are present in the figure legend, table legend, main text, or Methods section.

n/a	Confirmed	
☐	☒	The exact sample size (*n*) for each experimental group/condition, given as a discrete number and unit of measurement
☐	☒	A statement on whether measurements were taken from distinct samples or whether the same sample was measured repeatedly
☐	☒	The statistical test(s) used AND whether they are one- or two-sided<br>*Only common tests should be described solely by name; describe more complex techniques in the Methods section.*
☐	☒	A description of all covariates tested
☐	☒	A description of any assumptions or corrections, such as tests of normality and adjustment for multiple comparisons
☐	☒	A full description of the statistical parameters including central tendency (e.g. means) or other basic estimates (e.g. regression coefficient) AND variation (e.g. standard deviation) or associated estimates of uncertainty (e.g. confidence intervals)
☐	☒	For null hypothesis testing, the test statistic (e.g. *F*, *t*, *r*) with confidence intervals, effect sizes, degrees of freedom and *P* value noted<br>*Give P values as exact values whenever suitable.*
☒	☐	For Bayesian analysis, information on the choice of priors and Markov chain Monte Carlo settings
☒	☐	For hierarchical and complex designs, identification of the appropriate level for tests and full reporting of outcomes
☐	☒	Estimates of effect sizes (e.g. Cohen's *d*, Pearson's *r*), indicating how they were calculated

Our web collection on statistics for biologists contains articles on many of the points above.

Software and code

Policy information about availability of computer code

Data collection	Fiji was used to analyze signal intensity (mean gray area), cell area (manual measurement)
Data analysis	Graphpad Prism 8 was used for all statistical analysis

For manuscripts utilizing custom algorithms or software that are central to the research but not yet described in published literature, software must be made available to editors/reviewers. We strongly encourage code deposition in a community repository (e.g. GitHub). See the Nature Research guidelines for submitting code & software for further information.

Data

Policy information about availability of data

All manuscripts must include a data availability statement. This statement should provide the following information, where applicable:

- Accession codes, unique identifiers, or web links for publicly available datasets
- A list of figures that have associated raw data
- A description of any restrictions on data availability

Data is available upon request. All underlying graph data is submitted as Supplementary Data

Field-specific reporting

Please select the one below that is the best fit for your research. If you are not sure, read the appropriate sections before making your selection.

☒ Life sciences ☐ Behavioural & social sciences ☐ Ecological, evolutionary & environmental sciences

For a reference copy of the document with all sections, see nature.com/documents/nr-reporting-summary-flat.pdf

Life sciences study design

All studies must disclose on these points even when the disclosure is negative.

Sample size	A minimum of n=3 was used as generally accepted number of replicas in biological research. Where practically possible, sample size was increased
Data exclusions	No data was excluded
Replication	Key experiments were repeated and findings were reproducible, Biological replicas from different experiments were combined into one data set
Randomization	All scaffolds or cell culture dishes were randomly assigned to an experimental group
Blinding	No blinding was used. Analysis of western blot or image analysis could not be done blindly, because the differences between the different groups were obvious to the researchers, even without labeling.

Reporting for specific materials, systems and methods

We require information from authors about some types of materials, experimental systems and methods used in many studies. Here, indicate whether each material, system or method listed is relevant to your study. If you are not sure if a list item applies to your research, read the appropriate section before selecting a response.

Materials & experimental systems

n/a	Involved in the study	
☐	☒	Antibodies
☐	☒	Eukaryotic cell lines
☒	☐	Palaeontology
☒	☐	Animals and other organisms
☒	☐	Human research participants
☒	☐	Clinical data

Methods

n/a	Involved in the study	
☒	☐	ChIP-seq
☒	☐	Flow cytometry
☒	☐	MRI-based neuroimaging

Antibodies

Antibodies used	All used antibodies are described in detail in the paper
Validation	Antibodies were validated with westernblot (band at the right height) and immunofluorescence (obtaining the expected staining pattern), on top of validations done by the manufacturer

Eukaryotic cell lines

Policy information about cell lines

Cell line source(s)	Cell types used are described in detail in the paper
Authentication	Authentication of the MSCs was done by the manufacturer and in house, based on trilineage differentiation. Authentication of fibroblasts and MG63s were done by the manufacturer
Mycoplasma contamination	All cells were regularly tested for mycoplasma and were always negative.
Commonly misidentified lines (See ICLAC register)	*Name any commonly misidentified cell lines used in the study and provide a rationale for their use.*
